# An introduction to implementation science for the non-specialist

**DOI:** 10.1186/s40359-015-0089-9

**Published:** 2015-09-16

**Authors:** Mark S. Bauer, Laura Damschroder, Hildi Hagedorn, Jeffrey Smith, Amy M. Kilbourne

**Affiliations:** Center for Healthcare Organization and Implementation Research, VA Boston Healthcare System and Department of Psychiatry, Harvard Medical School, Boston, MA USA; Center for Clinical Management Research, VA Ann Arbor Healthcare System, Ann Arbor, MI USA; Substance Use Disorder Quality Enhancement Research Initiative and Center for Chronic Disease Outcomes Research, Minneapolis VA Health Care System & Department of Psychiatry, University of Minnesota School of Medicine, Minneapolis, MN USA; Mental Health Quality Enhancement Research Initiative, Central Arkansas Veterans Healthcare System, North Little Rock, AR USA; VA Quality Enhancement Research Initiative (QUERI), VA Office of Research and Development, Washington, DC USA; Department of Psychiatry, University of Michigan, Ann Arbor, MI USA; VA Boston Healthcare System, 152M, 150 South Huntington Avenue, Jamaica Plain, MA 02130 USA

**Keywords:** Implementation, Quality Improvement, Health Services, Outcome Assessment, Evidence-Based Practice, Learning Healthcare Organization

## Abstract

**Background:**

The movement of evidence-based practices (EBPs) into routine clinical usage is not spontaneous, but requires focused efforts. The field of implementation science has developed to facilitate the spread of EBPs, including both psychosocial and medical interventions for mental and physical health concerns.

**Discussion:**

The authors aim to introduce implementation science principles to non-specialist investigators, administrators, and policymakers seeking to become familiar with this emerging field. This introduction is based on published literature and the authors’ experience as researchers in the field, as well as extensive service as implementation science grant reviewers.

Implementation science is “the scientific study of methods to promote the systematic uptake of research findings and other EBPs into routine practice, and, hence, to improve the quality and effectiveness of health services.” Implementation science is distinct from, but shares characteristics with, both quality improvement and dissemination methods. Implementation studies can be either assess naturalistic variability or measure change in response to planned intervention. Implementation studies typically employ mixed quantitative-qualitative designs, identifying factors that impact uptake across multiple levels, including patient, provider, clinic, facility, organization, and often the broader community and policy environment. Accordingly, implementation science requires a solid grounding in theory and the involvement of trans-disciplinary research teams.

**Summary:**

The business case for implementation science is clear: As healthcare systems work under increasingly dynamic and resource-constrained conditions, evidence-based strategies are essential in order to ensure that research investments maximize healthcare value and improve public health. Implementation science plays a critical role in supporting these efforts.

## Background: what is implementation science and how did it develop?

### The need for a science of implementation

It has been widely reported that evidence-based practices (**EBPs**) take on average 17 years to be incorporated into routine general practice in health care [1–3]. Even this dismal estimate presents an unrealistically rosy projection, as only about half of EBPs ever reach widespread clinical usage [1].

Historically, this research-to-practice gap has not been the concern of academic clinical researchers. The traditional academic business case for career success has primarily supported conducting descriptive or mechanism-oriented studies and intervention studies on highly selected, typically academic medical center-based populations, and publishing in—ideally—top quality academic journals. Whether these findings translate into public health impact has typically not been the concern of traditional healthcare researchers.

This paradigm for academic success has justly come under criticism in recent years. Payers for biomedical research have been concerned over the lack of public health impact of their research dollars [4]. Moreover, decreasing research funding world-wide has led to debates over the trade-offs between investing in more conservative projects with predictable results versus more innovative research, including projects involving more real-world samples that could result in greater public health impact [5].

Recognition of the need for research that more directly impacts public health has broadened the academic mindset somewhat, from an exclusive emphasis on efficacy studies to more broadly generalizable effectiveness trials (Table 1) [6]. Several overlapping names, and conceptual structures, have developed for these latter types of trials including “effectiveness trials” [7, 8] “pragmatic clinical trials” [9]“practical clinical trials” [10] and “large simple trials” [11].

**Table 1:** Characteristics of Efficacy vs. Effectiveness Trial Designs (after [8])

	Efficacy Trial	Effectiveness Trial
Validity Priority	Internal > External	External ≥ Internal
Population and Sample	• Highly selected for condition of interest, narrowly defined	• Selected for condition of interest, reflecting presentation in source population
	• Few comorbidities	• Comorbidities resemble those in population to which results will be applied; only those who cannot practically or ethically participate are excluded
	• Willing and motivated participants	
Intervention	• Intervention staff are highly qualified	• Staff selection, training, and fidelity monitoring resemble those likely to be feasible in target sites outside of the protocol proper
	• Training may be intensive	
	• Fidelity monitoring may be similarly intensive	
Outcome Measures and Data Collection	• Outcome measurements can be extensive, casting a wide net for potential secondary effects, moderators and mediators, or adverse effects	• Outcome batteries minimize respondent burden (in terms of both frequency and length of assessments) since subjects are heterogeneous in their willingness and capability to participate
	• Since subjects are motivated, respondent burden less of a concern	• Accordingly, outcome measures chosen carefully to target fewer outcomes, and must be simple to complete
Data Analysis	• Standard statistical approaches suffice, and data-intensive analyses may be feasible	• Analyses to account for greater sample heterogeneity
		• Analyses account for more missing data and data not missing at random

However, such trials in and of themselves provide little guarantee of public health impact. Effectiveness trials typically depend, like efficacy trials, on a research resources which are separate from the clinical infrastructure and which are externally funded, time-limited, and evanescent at the end of the protocol [8]. Research-supported resources seldom remain at local sites to support continued use of even successful interventions; there is typically little institutional memory for the intervention, and no technology transfer. Moreover, EBPs often have characteristics that make unassisted translation into practice unlikely, especially if the intervention requires incorporating a change in a clinician’s routine [12].

A useful conceptualization of the biomedical research process has been as a “pipeline” [13] whereby an intervention moves from efficacy through effectiveness trials to sustained application in general practice. Blockages can appear at various stages, leading to quality gaps as EBPs are applied in less controlled settings. Many factors can impede EBP uptake, including competing demands on frontline providers; lack of knowledge, skills and resources; and misalignment of research evidence with operational priorities can all impede uptake. Accordingly, there is clear need to develop specific strategies to promote the uptake of EBPs into general clinical usage [14]. Implementation science has developed to address these needs.

### The organization of this summary

The aim of this review is to introduce non-specialist investigators, administrators, and policymakers to the principles and methods of implementation science. We compiled this review based on published literature, our experience as practicing implementation researchers, and extensive service on implementation science grant review committees. We include examples to illustrate critical principles, utilizing primarily studies of psychosocial EBPs. Most of these examples come from studies funded by the US Department of Veterans Affairs (USVA), since we are able to present these cases with a look “behind the scenes” to provide insights that may not be evident from simply reading the published results. However, it is important to note that the field of implementation science is a global field, as perusal of some of the major publication venues will make evident, such as *Implementation Science*, *BMC Health Services Research*, and a variety of specifically themed journals.

In this review we first define implementation science, comparing and contrasting it to the methods of quality improvement and dissemination. We then trace the development of implementation science as a field, illustrating this trajectory with experience in the USVA. We then drill down into the field, beginning with the basics of descriptive methods, including the pivotal role played by theories, models, and frameworks. Next we introduce the fundamentals of controlled intervention trials, including the quickly developing area of hybrid effectiveness-implementation designs. We next provide a detailed description of two real-world implementation studies, and close by placing implementation science into the context of global health policy.

### Defining implementation science

Implementation science can be defined as “the scientific study of methods to promote the systematic uptake of research findings and other evidence-based practices into routine practice, and, hence, to improve the quality and effectiveness of health services” [15]. This field incorporates a scope broader than traditional clinical research, focusing not only at the patient level but also at the provider, organization, and policy levels of healthcare (Table 2). Accordingly, implementation research requires trans-disciplinary research teams that include members who are not routinely part of most clinical trials such as health services researchers; economists; sociologists; anthropologists; organizational scientists; and operational partners including administrators, front-line clinicians, and patients.

**Table 2:** Types of Studies to Address Blockages in the Implementation Process

Implementation Process Gap	Types of Studies
Limited external validity of efficacy/effectiveness studies	• Design clinical interventions ready for implementation earlier in the research pipeline, emphasizing tools, products, and strategies that mitigate variations in uptake across consumer, provider, and or organizational contexts
Quality gaps across systems due to variations in organizational capacity (e.g., resources, leadership)	• Assess variations and customize implementation strategies based on organizational context
	• Data infrastructure development to routinely capture or assess implementation fidelity, patient-level processes/outcomes of care, and value/return-on-investment measures
	• Further refinement of implementation strategies involving organizational and/or provider behavior change
	• Development of provider/practice networks to conduct implementation studies or evaluation of national programs
Frontline provider competing demands (e.g., multiple clinical reminders)	• Refinement of implementation strategies using cross-disciplinary methods that address provider behavior/organizational change (e.g., business, economics, policy, operations research. etc.)
	• Positive deviation or adaptation studies especially to improve implementation at lower-resourced, later-adopter sites
Misalignment with national or regional priorities	• National policy/practice roll-outs
	• Randomized evaluations of national programs or policies

### Implementation sciences, quality improvement, and dissemination

Both implementation science and quality improvement (QI) efforts share the ultimate goal of improving the quality of healthcare. Methods used in the two fields often overlap, although there are some differences. QI efforts usually begin with a specific problem in a specific healthcare system, recognized at the level of the provider, clinic, or health system, and lead to the design and trial of strategies to improve a specific problem for that specific healthcare system. A variety of QI approaches have developed, often taken from other industries such as Toyota Lean, Six Sigma, and others [16, 17].

In contrast, implementation science typically begins with an EBP that is under-utilized, and then identifies and addresses resultant quality gaps at the provider, clinic, or healthcare system level. Additionally, implementation science, as a science, takes as part of its mission an explicit goal of developing generalizable knowledge that can be widely applied beyond the individual system under study. Nonetheless, both approaches share a common goal, value system, desire for rigor, focus on outcomes, and many common methods.

Dissemination, in contrast to implementation, refers to the spread of information about an intervention, assisted at most by educational efforts [18]. Again, however, there is some overlap in that implementation efforts may incorporate dissemination techniques, but they are typically embedded in more comprehensive, targeted, and active efforts to spread the EBP.

### Development of implementation science

Over the past 10–15 years healthcare systems that carry responsibility for managing fixed budgets to care for their populations have become increasingly attentive to problems of implementation in order to maximize the value of their healthcare funds [19]. Such organizations include several countries’ national health services, and the USVA in the United States. More recently in the US, new laws such as the Affordable Care Act have led to the development of accountable care organizations, which align care providers, often across hospital systems, and which frequently manage care for a given population under a fixed or constrained reimbursement cap [20]. As a result, US federal research funding organizations have recently developed an investment in implementation science [21, 22]. Another North American program, Knowledge Translation Canada [23], strives to improve the impact of research findings through implementation science strategies.

Among the oldest and most extensive scientific programs focusing on implementation is the USVA’s Quality Enhancement Research Initiative (QUERI; [13]), which we provide as a case study in the development of implementation science as a field. Since its inception in 1998, the goal of QUERI has been to improve military veterans’ health by supporting the more rapid movement of effective interventions into practice [24]. QUERI combines scientific rigor provided by leading academic researchers with partnerships with USVA operations leaders who face pressing implementation issues. QUERI is uniquely positioned as an implementation science resource within a national healthcare system, and as a result provides significant academic-operations synergies: QUERI focuses on increasing the health system impact of EBPs of clinical and policy importance to VA operations, and through scientific rigor contributes foundational knowledge to the field of implementation science which can have impact well beyond the USVA system itself.

Reflecting the development of implementation science as a field, QUERI’s first evolutionary leap was its transition from investment in conventional health services research studies to research that focused on description of gaps in the use of EBPs and description of relevant barriers and facilitators. The second evolutionary leap, which is currently ongoing, is a transition from descriptive studies of barrier/facilitators to specifying and testing optimal strategies for implementation, including both controlled trials and observational studies that evaluate natural experiments of practice-based improvement initiatives. Most recently, QUERI’s mission has extended to support implementation of EBPs, as promoted by the US Office of Management and Budget [25] through the use of rigorous evaluation to study and scale-up programs or initiatives that have demonstrated effectiveness, or use randomized program design to test policies or programs at a population level [26, 27].

## Discussion: the principles and methods of implementation science

### Implementation Studies Differ from Clinical Studies

The first step in understanding implementation studies is to distinguish implementation processes from the EBPs they seek to implement. An *implementation intervention* is “a single method or technique to facilitate change,” while an *implementation strategy* is “an integrated set, bundle, or package of discreet implementation interventions ideally selected to address specific identified barriers to implementation success” [13]. Implementation interventions may include, for example, efforts to change behavior at the patient, provider, system, or even policy level. Common examples include strategies at the provider level such as education/training, audit-feedback, and performance incentives. Strategies targeting the provider, team, or clinic levels may include QI techniques or other systems redesign efforts, team-based performance incentives, learning collaboratives, or community-engagement. Facilitation, guided efforts by internal or external organizational staff to support multiple levels of system change through provider or team-based coaching, is increasingly recognized as critical to the success of many implementation effects [28, 29].

In contrast to clinical research, in which typically focuses on the health effects of an EBP, implementation studies typically focus on *rates and quality of use of EBPs* rather than their effects. Such EBPs may be as “simple” as increasing use of a single medication such as beta-blockers in individuals who have experienced a myocardial infarction or the use of metabolic side effect monitoring for individuals taking antipsychotic medications, or as complex as instituting psychotherapies like cognitive-behavioral therapy or even multi-component care paradigms such as the collaborative chronic care model [30].

While the distinction between the implementation strategy and the EBP seems clear in concept, careful attention to their distinct roles is imperative in developing focused hypotheses and making evaluation design decisions. For instance, in studying the effects of a program to increase effective cognitive-behavioral therapy use for bipolar disorder, the impact of cognitive-behavioral therapy on health status would be an EBP outcome (and more typically a focus for a clinical study), while measuring the proportion of clinicians providing cognitive-behavioral therapy, or proportion of patients who attend a minimum level of cognitive-behavioral therapy sessions would be a more typical implementation study outcome.

### Evaluating the implementation process and its impact

Thus the crux of implementation studies is their focus on evaluating the process of implementation and its impact on the EBP of interest. These studies can involve one or more of three broad types of evaluation, *process evaluation*, *formative evaluation,* and *summative evaluation* [13, 31].

*Process evaluation* simply describes the characteristics of use of an EBP (or lack thereof). Data are collected before, during, and/or after the implementation and analyzed by the research team without feedback to the implementation team and without intent to change the ongoing process. Process evaluation can be undertaken in a purely observational study (e.g., in preparation for developing an implementation strategy) or during the course of a spontaneous or planned system or policy change.

*Formative evaluation* utilizes the same methods as process evaluation, but differs in that data are fed back to the implementation team and/or staff in the target system during the study in order to adapt and improve the process of implementation during the course of the protocol. Formative evaluation is conceptually similar to fidelity monitoring that goes on as part of any traditional clinical trial, but differs in that it is specified *a priori* in a study hypothesis or research question.

A quantitative version of formative evaluation in clinical trials is the use of sequential multiple assignment randomized trials (SMART) or adaptive intervention designs, which are used to either augment or switch treatments at critical decision points where there is evidence of initial non-response [32, 33]. As we discuss below, the use of formative evaluation has implications for controlled trial validity, which may differ between clinical and implementation trials.

*Summative evaluation* is a compilation, at study end, of the impact of the implementation strategy. Summative evaluation measures typically assess impacts on processes of care (e.g., increased use or quality of targeted EBP). Another common component of summative evaluation is to characterize the economic impact of an implementation strategy and its effects. Implementation studies most commonly do not employ formal cost-effectiveness analyses but conduct focal “business impact analyses” [34]. Such analyses focus on estimating the financial consequences of adoption of a clinical practice within a specific healthcare setting or system. Typically this includes costs to the system associated with both the implementation strategy and the utilization of the EBP.

### Types of evaluation data

Data for the process, formative, and/or summative evaluation can come from various sources and can include either or both of quantitative and qualitative data. Data can be collected across various levels of observation including patients; providers; systems; and broader environmental factors such as community, policy, or economic indices.

Common *quantitative* measures include structured surveys and tools that assess, for example, organizational context, provider attitudes and behaviors, or patient receptivity to change. Administrative data are often utilized, either in focal target populations or at the broader system level to characterize, for example, baseline and change in rates of utilization of particular practices. Measures of fidelity to the EBP are often central components of the evaluation plan, and these can be quantitative, qualitative, or both.

Common *qualitative* data collection methods include semi-structured interviews with patients, providers, or other stakeholders; focus groups; direct observation of clinical processes; and document review. Qualitative data collection and analysis can either be structured as a hypothesis-free exploration using grounded theory or related approaches [35] or can utilize directed content analysis [36] to address pre-specified issues such as hypothesis-testing or measurement of intervention fidelity. Most implementation evaluation processes include mixed qualitative and quantitative measures, and require careful attention in the study design to the various ways to combine such data [37].

## The role of theory and its link to specific design decisions

### The need for theory-based implementation

Describing, implementing, and then sustaining any innovation is a complex undertaking [38] —complex because implementation strategies (a) are typically multi-component and (b) must adapt to local contexts. Contexts in which implementation efforts occur are themselves complex because of multiple interacting levels (e.g., patients, providers, teams, service units), with wide variation from setting to setting [39]. Without a thorough understanding of the context, an EBP may either not be adopted or may be taken up in an adapted form with compromised fidelity due to contextual pressures. The endeavor requires clear, collective, consistent use of theory to build knowledge about what works, where, and why [40].

### Theories, models, and frameworks

The terms *theory*, *model*, and *framework* are, unfortunately, often used interchangeably and imprecisely. Use of the term *theory* in this paper refers to any proposal about meaningful relationships between constructs (variables) or how a mechanism or construct may change the behavior of another construct or outcome [41, 42].

**Fig. 1:**
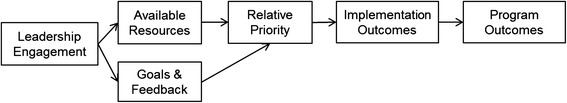
Model of a Hypothesized Pathway of Change

A theory may be operationalized within a *model*, which is a simplified depiction of a more complex world with relatively precise assumptions about cause and effect. For example, the model in Fig. 1 depicts a single hypothesized pathway of change for implementing a weight management program [43, 44]. This model hypothesizes that leadership engagement will result in sufficient resources for implementation, and in leaders’ articulating implementation goals that align with organizational priorities. These leadership actions serve to raise organizational perceptions of priority, which in turn leads to positive implementation outcomes (e.g., high fidelity of the program to its intended design), and thus to better weight loss for patients. Each construct in the model can be assessed and the model tested to affirm or disprove this pathway of change. Based on results, the model may need refinement or be rejected altogether.

*Frameworks* provide a broad set of constructs that organize concepts and data descriptively without specifying causal relationships. They may also provide a prescriptive series of steps summarizing how implementation should ideally be planned and carried out [13, 45]. For example, the Consolidated Framework for Implementation Research (CFIR, [46]) classifies 39 implementation constructs across five domains considered to be influential moderators or mediators of implementation outcomes, providing a structure by which to systematically assess context within which implementation occurs [47]. Optimally, researchers will also circle back and utilize empirical data to assess the usefulness of the guiding theories, models, or frameworks used to design the study and offer recommendations to improve them [48]. Consistent use of constructs across studies further allows more efficient syntheses through use of e.g., qualitative comparative analyses techniques [49]. Explicit operationalization of theoretical constructs can also guide development of robust quantitative measures [50, 51].

### Example: using a framework to guided data collection and analysis

An example will illustrate the role of such frameworks. Damschroder and Lowery conducted an evaluation of a weight management program in USVA, funded by the QUERI program described above [43, 44]. There was wide variation in program implementation 1.5 years after initial dissemination. CFIR was used to develop a guide to interview staff at local facilities to understand what contributed to this variation. Quantitative ratings were applied to qualitatively coded interview data [36] and used to identify constructs that distinguished between sites with better versus worse implementation outcomes. Ten constructs were found to be related to successful implementation. For example, the sites with the poorest implementation outcomes had lower ratings for “Goals and Feedback” because staff at these sites did not have the time or skills to identify program goals or monitor progress toward those goals. This information can be used to develop more specific models to guide future implementations and identify discrete implementation strategies (e.g., audit-feedback [52], identify champions [53]).

## Controlled implementation trials

### Types of controlled implementation trials

Many implementation studies seek to identify barriers and facilitators of EBP adoption under naturalistic conditions. However, other studies seek to enhance EBP adoption by employing specific implementation strategies in controlled trials. Controlled implementation trial designs are of two major types: parallel group and interrupted time series. *Parallel group designs* are randomized and prospective, similar to other types of health services or clinical trials. The unit of randomization depends on the strategy and the outcome of interest, and may be the patient, provider, clinic, facility, or system level. Some creative designs respond to real-world constraints by combining characteristics of historical control studies with true matched randomization [28]. Additional innovative designs have been employed, such as stepped wedge designs in which all participating sites receive implementation support, though staggered in time [54, 55], resembling traditional incomplete block designs. Additionally, controlled implementation trials sometimes randomize a small number of sites and measure outcomes at the individual patient level, utilizing multivariate nested analyses [56]. Finally, SMART randomization designs, as mentioned above [32], can bring an added degree of rigor to the use of within-study formative evaluation.

In the real world of policy roll-outs and program changes, parallel group designs may not be feasible for pragmatic, political, or ethical reasons. In such situations, *interrupted time series designs* can be utilized. These also resemble their counterparts among health services and clinical trials, optimally with the implementation outcome of interest measured at multiple time points prior to and after an implementation effort. Such designs are most feasible when the implementation strategy is a discrete event, such as a policy change [57] since more typical implementation strategies exert their effect over months, making the “interruption” in the time series more difficult to pinpoint.

### Implementation versus traditional health services and clinical trials

Controlled implementation trials differ from other types of health services and clinical trials in two major ways. First and most basically, as noted above, they focus on the impact of the implementation strategy on the use of an EBP, rather than health impact of the EBP itself. Second, they take a fundamentally different approach to validity, as illustrated below.

Consider a hypothetical trial of motivational interviewing (**MI**) for substance use disorders in the homeless population (Table 3). Implementation designs differ from the intervention trials at a basic level, that of the *hypothesis*. While intervention trials focus on comparing the intervention to a comparison group, implementation trials focus on the ability of the research team to increase adoption of MI. While the *population and setting* may resemble that for effectiveness trials, the unit of observation in implementation trials may be providers or clinics—depending on the focus of the implementation efforts. As the population becomes more heterogeneous, *outcome measures* must become briefer and simpler in order to minimize the respondent burden and retain in the protocol subjects who are less research-tolerant. As one moves from left to right in Table 3, *intervention clinicians* become progressively less highly specialized and skill development efforts more closely resemble training that would typically be feasible under clinical conditions; measurement of fidelity to the intervention varies similarly.

**Table 3:** Intervention vs. Implementation Trial Design Perspectives: A Hypothetical Example of the Use of Motivational Interviewing (MI) for Substance Use Disorders in the Homeless Population

	Efficacy Design Principles	Effectiveness Design Principles	Implementation Design Principles
Hypothesis	MI beats control	MI beats control	MI will be adopted and sustained
Population & setting	Exclude psychosis, bipolar, anxiety; any setting with cooperative patients	Include most comorbidities; typical setting is nonspecialized practice sites	Unit of observation may be patients, providers, or clinics; typical setting is nonspecialized practice sites
Outcome measures	Health outcomes, many: "just in case…"	Health outcomes, short & sweet	Emphasize MI adoption measures
Intervention: clinicians	PhDs, MSWs hired & trained by PI	Addiction counselors hired as study staff	Endogenous addiction counselors
Intervention: fidelity	Trained to criterion, audiotaped for fidelity	Trained to criterion, QI-type monitoring as in clinical system	Formative evaluation the focus
Context	Make sure that the trial is successful, at all costs	Work within “typical” conditions	Maintain typical conditions
Research support	Crypto-case management	Research support, but “firewalled”	Research support limited; e.g., only for training
Validity emphasis	Internal > > external	External > internal	Plan to optimize protocol in real time using formative evaluation, in violation of “traditional” considerations of internal validity, while systematically documenting adaptations

The consideration of the *context* for the research also differs substantively. Efficacy trials value conducting a trial that is invariant from beginning to end. This entails sometimes extra-ordinary efforts at training clinicians and tracking subjects, so that the research team’s efforts amount at times to “crypto-case management”—which is not a problem since the focus of the trial is solely on testing the intervention under optimal conditions. However, since effectiveness studies are more concerned with performance of an efficacious intervention in real-world settings, careful attention is paid to limiting the amount of research-funded effort that is incorporated into the protocol, often “firewalling” research effort with carefully and explicitly defined parameters (e.g., 8). Further, for implementation trials, trying to optimize the natural context in which adoption is being measured is a threat to validity of the trial. Thus researcher involvement in the site of adoption is drastically circumscribed, with some implementation trials even limiting research support to training endogenous clinicians and utilizing remote, “light-touch” outcome assessments for patient and provider subjects.

These differences are driven by radically different priorities regarding trial *validity* [58]. *Internal validity* prioritizes establishing a causal connection between the intervention and the outcome; in the service of this aim, sample, outcome measurements, intervention are all highly controlled without consideration of applicability to other considerations outside of the trial itself. *External validity* prioritizes the generalizability of trial results to other relevant populations and situations. While efficacy studies value internal over external validity, the balance shifts for effectiveness studies: Although the effectiveness trial must have sufficient internal validity to carry out the study successfully, explicit design decisions are made that value generalizability beyond the trial itself.

Finally, implementation trials, in contrast to both types of intervention trials, aim at arriving at the optimal implementation strategy, and maximal EBP impact, by the end of the study, rather than isolating specific mechanisms. Note, however, that in subtle ways this is the case for *all* types of studies, including efficacy trials, in which intervention fidelity data are assiduously collected throughout the study and adjustments in training and even personnel are made to ensure internal validity. The difference with implementation trials is that such modifications are planned for through formative evaluation, and often specifically hypothesized about [31].

### Hybrid effectiveness-implementation trial designs

Although an implementation research protocol can demonstrate improvements in the uptake of EBPs, the impact of such practices on health outcomes has not classically been a concern of implementation science. However, it is clear from many studies that the assumption that improved processes mean improved health outcomes is flawed [59, 60]. Additionally, even if EBPs are implemented as planned with adequate fidelity, there may be a “voltage drop” as an EBP moves from the effectiveness clinical trials setting into implementation in real-world clinical practices. Moreover, it is important to know the degree to which an EBP, when utilized in general clinical practice, retains the active mechanisms while adapting to local site needs and priorities [61, 62].

Cognizant of these needs, a group of implementation and clinical trial researchers codified the concept of *hybrid effectiveness-implementation designs* [63]. “Codify” is the correct term, rather than “develop,” since all the concepts and methodologies involved in hybrid designs already existed across often disparate scientific fields; the purpose of the hybrid design concept paper was to bring together those concepts and methodologies into a single framework and explore the utility of the framework for the needs of real-world researchers and public health stakeholders. In a few short years this framework has begun to be adopted by researchers and funders [64].

Hybrid research designs are defined as trial designs that take a dual focus *a priori* of assessing both the effectiveness of the implementation strategy in enhancing the use of the EBP and the health impact of the EBP [63]. Thus hybrid designs pose hypotheses regarding both EBP and implementation effects, typically measuring both healthcare processes (implementation outcomes) and health status (intervention outcomes). Not all hybrid designs apply controlled trial methodologies to both aspects of outcome simultaneously, but all incorporate *a priori* the assessment of both of these domains explicitly during the development of the research design. There are three types of hybrid designs.

*Hybrid Type I* designs test the health impact of an EBP while explicitly collecting data on the implementation process to facilitate subsequent implementation efforts. Such designs are typically utilized preliminary to an implementation trial. Some advocates say that all effectiveness trials should be Hybrid Type Is.

*Hybrid Type III* designs test the ability of an implementation strategy to enhance use of an EBP while collecting data on health impact of the EBP during implementation. These designs are useful when the effectiveness of an intervention is well established, but it is unclear how robust the effects are likely to be under implementation conditions. Some advocates say that all implementation trials that involve processes to improve health outcomes should be Hybrid Type IIIs.

*Hybrid Type II* designs are a hybrid of hybrids: They test both the EBP effects on health outcome and the implementation strategy effects on EBP use. Such designs are utilized when there is substantial “implementation momentum” within a clinical system, but intervention effectiveness data are somewhat less extensive or only indirect. Thus both the impact of the implementation strategy on EBP usage and the impact of the EBP on health status are the dual foci of Hybrid Type II designs.

## Two real-world implementation study examples

A brief description of two USVA QUERI projects will illustrate some of the concepts covered in this review.

### Incentives in substance use disorders (Illustrating a type I hybrid design)

The first exemplar [65, 66] is an effectiveness trial with an observational implementation component fully integrated into its design (Hybrid Type I). The rationale behind adding the implementation component to this trial was that if the intervention proved to be effective, information on the most feasible strategies for implementation and the most significant barriers likely to be encountered would already be collected, informing future implementation efforts. As the methods of the randomized, controlled effectiveness trial portion of the study are standard and familiar to most researchers (see also Table 3), this description will focus on the process evaluation. The process evaluation was guided by two frameworks. RE-AIM [67] guided the investigator to examine the *R*each, *E*ffectiveness, *A*doption, *I*mplementation and *M*aintenance of the intervention, posing questions such as how many patients will be interested in receiving the intervention, what information will a clinic require to consider adoption of the intervention, which training, tools and/or resources will the clinic require to implement the proposed intervention with high fidelity and what resources will be required for the clinic to maintain the intervention long-term. In complementary fashion, the PARIHS framework (*P*romoting *A*ction on *R*esearch *I*mplementation in *H*ealth *S*ervices [68]; recently revised [69]) proposes that successful implementation is a function of the strength of the *evidence* supporting the intervention, the *context* in which the intervention is implemented and the *facilitation* provided to support implementation. These constructs guided the investigators to determine how the evidence base for the intervention is perceived by practitioners, which characteristics of the context impeded or facilitated implementation, and which tools and supports facilitated dealing with identified barriers.

The process evaluation for this study combined multiple data collection tools and strategies in an attempt to identify key barriers and facilitators related to each of these constructs. Data collection tools included administrative data, patient surveys, staff surveys and interviews, clinic leadership interviews and cost tracking data. Information from these multiple sources allowed the identification of key barriers and facilitators likely to be encountered in any efforts to implement the incentive intervention in additional clinics and allowed for the creation of recommended implementation strategies (Table 4).

**Table 4:** Enhancing Implementation of Incentive Therapy for Substance Use Disorders Guided by the RE-AIM & PARIHS Frameworks (Section VI.A)

RE-AIM Framework [67]	
Reach	• Target intervention to patients that will be attending treatment at least twice • per week for other treatment services.
Adoption	• Solicit explicit support from the highest levels of the organization through, for example, performance measures or treatment recommendations.
	• Identify or create measures of clinic effectiveness which can be used to identify gaps in performance and monitor the impact of implementation.
	• Solicit agreement in advance for designated funding.
	• Educate leadership about potential strategies for integrating the intervention into current practices.
	• Adopt incrementally. Start with a specific treatment track or clinic to reduce staff and funding burden until local evidence of effectiveness and feasibility is available to support spread
Implementation	• Train staff on urine test cups and breathalyzer including sensitivity and specificity of the screen results.
	• Make scripts available for communicating positive and negative test results to patients.
	• Supply a tracking database to ensure consistency in awarding prize picks.
	• Provide a step by step intervention appointment protocol.
	• Facilitate documentation in the electronic health record.
Maintenance	• Ensure all staff are aware of their responsibilities related to incorporating information from the intervention into clinical interactions with patients to facilitate integration into the clinic.
	• Consider option of having case managers administer the intervention to their own patients rather than having one or two individuals responsible for the intervention.
PARIHS Framework [68, 69]	
Evidence	• Staff may not be aware of strength of evidence or may express philosophical disagreement with incentive interventions: Engage staff early on to understand and address concerns.
	• Staff may need education on evidence and/or how behavioral reinforcements function in a variety of settings.
	• Staff may benefit from engaging with clinics that have already implemented or may be willing to engage in a brief test of the intervention.
Context	• Even in highly supportive contexts, barriers are substantial and implementation has a high likelihood of failure if barriers are not identified and addressed up front.

### Implementing pharmacotherapy for alcohol use disorders (illustrating formative evaluation)

The second example is an ongoing implementation trial that uses formative evaluation techniques to refine and test a theory-driven implementation strategy to increase access to alcohol use disorder pharmacotherapy in primary care settings. The rationale for including the formative evaluation was that no matter how carefully an implementation strategy is planned in advance, the experience of actual implementation leads to insights which can be utilized to improve the strategy even further. The development of the initial strategy was based on prior formative QUERI work as well as the Theory of Planned Behavior [70]. Earlier qualitative interviews with providers had documented: (1) lack of provider confidence in medication effectiveness, (2) lack of provider skills and knowledge about using the medications, and (3) perceived low patient demand as the top three barriers to implementation. This work suggested that implementation interventions would have to focus not only on education but also on “marketing” and that the interventions would need to focus not only on providers but also on patients.

The Theory of Planned Behavior was used as a guiding theory because the desired behavior change is primarily at the individual level, e.g., convincing providers and patients to consider pharmacotherapy as a viable intervention option for alcohol use disorders. The Theory of Planned Behavior states that behavioral intentions are guided by attitudes toward the behavior, subjective peer norms, and perceived behavioral control and implementation interventions were selected to address each of these constructs. Implementation strategies selected for providers include social marketing, access to peer expert consultants, feedback on prescribing rates, and education and decision support tools. Direct-to-consumer mailings were selected as the intervention to address lack of patient awareness of medication options and potential negative peer attitudes toward pharmacological treatment of alcohol use disorder and to encourage patients to discuss alcohol use with their primary care provider. The implementation strategy is being evaluated using an interrupted time-series design with controls. Prescribing rates are being monitored for 9-month pre-implementation, implementation, and post-implementation phases for intervention facilities and matched control facilities. Already in the study, formative evaluation has led to refinements of the implementation strategy (Table 5).

**Table 5:** Developmental Formative Evaluation: The Example of Pharmacotherapy for Alcohol Use Disorders (Section VI.B)

Key Barriers Identified (Exemplar Quote)	Resulting Implementation Refinements
Lack of time: “I don’t have time for this. Patients are not coming in asking to address their alcohol use. I’d be lucky to have 5 min to discuss this.”	• Condense educational materials.
	• Make materials available through link in computerized record provider will already have open.
	• Present research demonstrating that brief conversations can reduce patient drinking levels.
Do not perceive current system as problematic: “The system works fine as it is. We have great substance use disorder folks to refer them to.”	• Data to demonstrate that majority of patients do not follow through on referrals.
	• Education on patient perspective: Patients that are not comfortable going to “addiction” or “mental health” clinic may address alcohol use as part of overall plan for improving health.
Perceived lack of patient interest: “Patients are not going to be happy with me if I bring this up. They don’t want to talk about it.”	• Present patient interview results: Patients want more information and would accept this information from their primary care provider.
Lack of proficiency: “I was never trained to deal with addictions. This is not in my purview.”	• Comprehensive education and consultation from peer specialists.
	• Stress impact of alcohol use on major diseases that they struggle to manage on a daily basis.

## Summary

Healthcare environments around the world are increasingly dynamic, resource-constrained, and interconnected—and are driven by equally complex political and economic environments. Accordingly, maximizing healthcare value [19] has become a policy imperative globally. Healthcare sciences must evolve in parallel to serve this need.

To this end, implementation science is becoming a critical tool for promoting what the US Institute of Medicine has called the “Learning Healthcare System.” The learning healthcare system can be defined as a healthcare system that employs a set of ongoing processes to provide higher value healthcare through systematic review of health care data analytics, and application of such data to inform promotion of overall strategies and targeted improvement efforts [71, 72].

To date, the bulk of attention to learning healthcare systems has focused on the acquisition and analysis of large datasets gathered from real-world clinical practices in as close to real-time as possible [73]. However, such data-gathering represents only the input arm of the learning healthcare organization. Healthcare systems also need to act on such data to improve practice. Implementation science provides a systematic set of principles and methods by which to accomplish this.

Provider and system behavior do not change by themselves, and unimodal interventions do not typically produce effective or lasting changes in systems as complex as healthcare [39]. Without concerted attention to evidence-based implementation strategies, learning healthcare systems risk developing massive, expensive repositories of information without adequate strategies to actually utilize such data for system change. As with earlier assumptions that mere publication of an efficacy or effectiveness trial results would lead to automatic EBP adoption, the assumption that comprehensive, elegant, real-time datasets will, in and of themselves, lead to practice change is at best an optimistic aspiration and at worst a massively expensive error.

Rather, sophisticated data-gathering systems must be paired with equally sophisticated implementation strategies if learning healthcare systems are to make good on their promise. The emerging science of implementation provides a systematized approach to identifying and addressing barriers and facilitators to system change, and thus represents a critical component of any learning healthcare system.

## Availability of data and materials

Not applicable.

## Abbreviations

CFIR: Consolidated Framework for Implementation Research
EBP: Evidence-based practice
MI: Motivational interviewing
NIH: United States National Institutes of Health
PARIHS: Promoting action on research Implementation in health services (an implementation framework)
QUERI: Quality Enhancement Research Initiative
QI: Quality improvement
RE-AIM: Reach, effectiveness, adoption, implementation, and maintenance (an implementation framework)
SMART: Sequential multiple assignment randomized trials
US VA: United States Department of Veterans Affairs, Veterans Health Administration
